# Primary myelofibrosis evolving to an aplastic appearing marrow

**DOI:** 10.1002/ccr3.1618

**Published:** 2018-05-31

**Authors:** Jordan K. Schaefer, Sarah M. Choi, Gary D. Luker, Thomas L. Chenevert, Brian D. Ross, Moshe Talpaz

**Affiliations:** ^1^ Division of Hematology/Oncology Department of Internal Medicine University of Michigan Ann Arbor MI USA; ^2^ Department of Pathology University of Michigan Ann Arbor MI USA; ^3^ Department of Radiology Center for Molecular Imaging University of Michigan Ann Arbor MI USA; ^4^ Department of Biomedical Engineering University of Michigan Ann Arbor MI USA; ^5^ Department of Microbiology and Immunology University of Michigan Ann Arbor MI USA; ^6^ Department of Biological Chemistry University of Michigan Ann Arbor MI USA

**Keywords:** aplasia, myelopoiesis, neoplasia, pathology, primary myelofibrosis

## Abstract

Our case highlights a series of bone marrow biopsies from a patient with primary myelofibrosis. Over time, this patient developed an unusual fatty appearance to his marrow, confirmed on multiple biopsies. This finding was supported by a quantitative fat MRI sequence that also shows a fatty appearance to the marrow.

## 1 CASE

**Figure 1:**
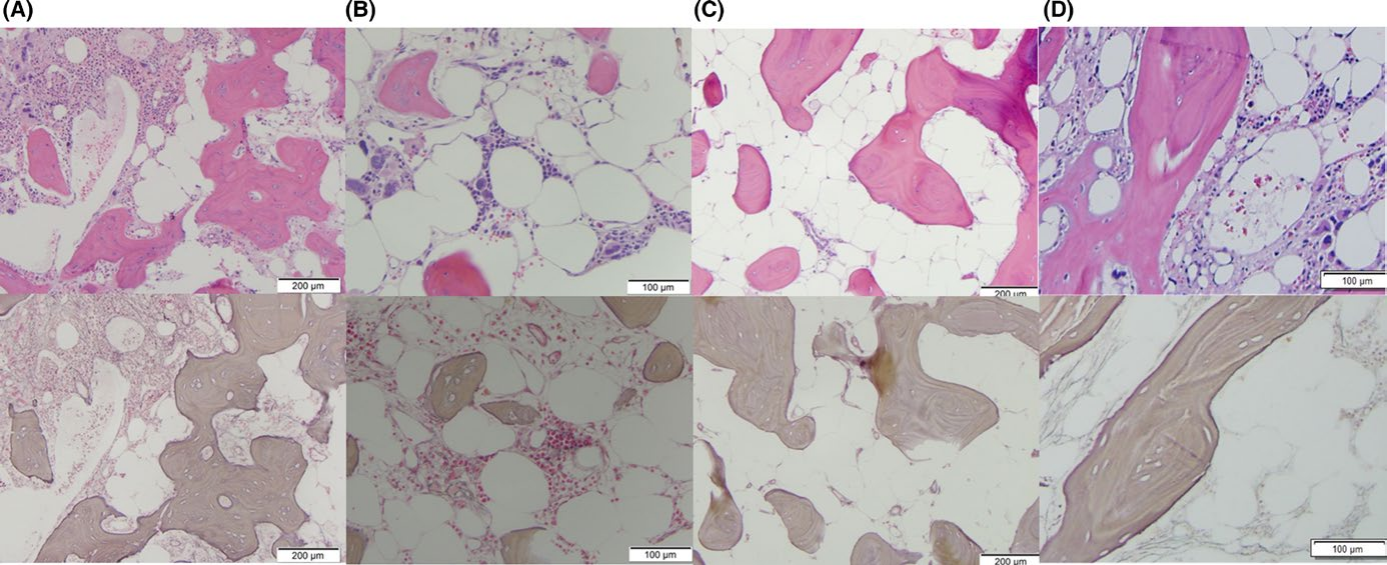
Histopathological findings from sequential bone marrow biopsies (panels A‐D) of a patient with primary myelofibrosis that ultimately developed fatty replacement of his marrow. (H&E, top panels; Reticulin, bottom panels)

**Figure 2:**
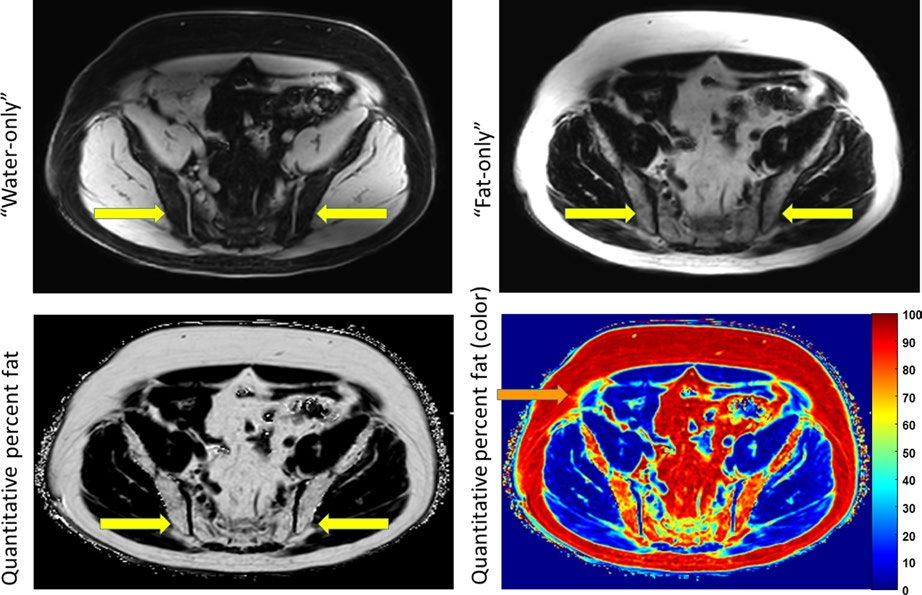
Quantitative fat MRI shows replacement of bone marrow with fat. (Top row) Representative transaxial image of the pelvis shows water‐only (left) and fat‐only (only) acquisitions from a quantitative fat imaging sequence. Yellow arrows show iliac crests. (Bottom row) Images display the percent fat in each voxel with a grayscale display (right) or pseudocolor display that depicts 100% fat and 0% fat as red and dark blue, respectively (left). Percent fat in bone marrow approximates values measured in subcutaneous fat (orange arrow)

A 54‐year‐old man was diagnosed with *MPL* mutated, primary myelofibrosis in 2004 with a hypercellular marrow, MF‐2 fibrosis with megakaryocytic atypia, peripheral leukoerythroblastosis, splenomegaly, and an elevated lactate dehydrogenase. He was observed until 2010, when he was started on hydroxycarbamide for increasing symptoms. Prior to his 2011 enrollment in a clinical trial utilizing a novel JAK2 inhibitor, fedratinib, a bone marrow biopsy (Figure 1, Panel A; hematoxylin and eosin stain above, reticulin stain below, 10×) showed progressive megakaryocytic atypia and MF‐3 fibrosis. Nearly 1 year into the trial, a repeat bone marrow (Figure 1, Panel B; hematoxylin and eosin stain above, reticulin stain below, 20×) was hypocellular with MF‐3 fibrosis only in the cellular areas. The patient was taken off trial in 2013 and started on ruxolitinib. Repeat bone marrow biopsies in 2016 (Figure 1, Panel C; hematoxylin and eosin stain above, reticulin stain below, 10×) show a marrow comprised entirely of adipose tissue with osteosclerosis. Quantitative fat magnetic resonance (MR) imaging, utilizing methods reported separately,^1^ shows replacement of the marrow with fat. Representative transaxial MR images of the pelvis show water‐only, and fat‐only (Figure 2, top row) constituents. Arrows show the iliac crests. A representative pseudocolor display that depicts 100% fat as dark red and 0% fat as dark blue is shown (Figure 2, bottom row). The percent fat evident in the bone marrow imaging approximates that in the subcutaneous fat. After 3 bone marrow biopsies and the MR demonstrating this unusual transition of primary myelofibrosis to a fatty, aplastic appearing marrow, a repeat bone marrow in 2017 (Figure 1, Panel D; hematoxylin and eosin stain above, reticulin stain below, 20×) now showed a hypocellular marrow with histopathologic findings consistent with his marrow at the time of diagnosis. To our knowledge, this is the first case of a fibrotic marrow developing such an aplastic appearance in a patient treated with a JAK2 inhibitor.

## AUTHOR CONTRIBUTION

JKS and MT: compiled the clinical information and wrote the manuscript. SMC: provided the photomicrographs and descriptions of the bone marrow biopsies. GDL, TLC, and BDR: provided the MRI images and descriptions. GDL, TLC, BDR, MT: developed and wrote IRB amendments to include advanced MRI protocols. All authors reviewed and approved the manuscript.

## CONFLICT OF INTERESTS

Dr. Talpaz is on the advisory board for CTI Biopharma and Gilead. He has received travel support from Ariad. The remaining authors have no relevant conflicts of interest.
